# Is the lockdown important to prevent the COVID-19 pandemic? Effects on psychology, environment and economy-perspective

**DOI:** 10.1016/j.amsu.2020.06.010

**Published:** 2020-06-14

**Authors:** Abdulkadir Atalan

**Affiliations:** Department of Industrial Engineering, Gaziantep Islam, Science and Technology University, 27010, Gaziantep, Turkey

**Keywords:** COVID-19, Lockdown, Correlation, Psychology, Environment, Economy

## Abstract

COVID-19's daily increasing cases and deaths have led to worldwide lockdown, quarantine and some restrictions. This study aims to analyze the effect of lockdown days on the spread of coronavirus in countries. COVID-19 cases and lockdown days data were collected for 49 countries that implemented the lockdown between certain dates (without interruption). The correlation tests were used for data analysis based on unconstrained (normal) and constrained (Tukey-lambda). The lockdown days was significantly correlated with COVID-19 pandemic based on unconstrained (r = −0.9126, F-ratio = 6.1654; t-ratio = 2.40; prob > .0203 with 49 observations) and based on Tukey-lambda (r = 0.7402, λ = 0.14). The lockdown, one of the social isolation restrictions, has been observed to prevent the COVID-19 pandemic, and showed that the spread of the virus can be significantly reduced by this preventive restriction in this study. This study offers initial evidence that the COVID-19 pandemic can be suppressed by a lockdown. The application of lockdown by governments is also thought to be effective on psychology, environment and economy besides having impact on Covid-19.

## Highlights

• The study examines the impact of lockdown (curfew) on the spread of Covid-19 pandemic.
• The correlation tests are used for data analysis.
• This study offers initial evidence that the COVID-19 pandemic can be suppressed by a lockdown.
• This study assesses the effects of lockdown on psychological, environmental and economy.

## 1 Introduction

A disease similar to pneumonia cases began to emerge in Wuhan City, Hubei Province, China in December 2019 [1,2]. The studies revealed that the cases that emerged were a new type of coronavirus that was not previously described. This form of the virus was called Coronavirus 2019, or COVID-19, since it appeared in 2019 [3]. The source of this virus is thought to be the Huanan seafood market in Wuhan, China. It was understood in time that the virus, which is transmitted from animal to human, can spread from human to human.

Although the molecular mechanism of COVID-19 transmission pathway from human to human is still not resolved, the principle of transmission of respiratory diseases is similar in general [4]. Respiratory diseases are spread by droplet scattering. In this type of spreading, a sick person is exposed to this microbe to people around him by coughing or sneezing. In other words, environmental factors play an important role in the transmission of this virus [5].

The COVID-19 outbreak is spreading very fast every day and more than 4 million people have been actively infected by this virus so COVID-19 restrictions are applied in almost all areas of life [6]. The most basic measure to reduce the spread of coronavirus or to prevent infection is to follow hygiene rules [7]. The most important of these is washing hands. For this reason, the spread of this virus is slower in societies that have the habit of washing hands and pay attention to the general hygiene rules [8]. There is a high level of participation in the "stay at home" call by official institutions. Scientists warn that the COVID-19 virus can reach any age group quickly [1,9].

Approximately 214 countries reported the number of confirmed COVID-19 cases [10]. Countries have taken very strict restrictions such as vacation for schools, working from home, quarantine for regions with high number of cases, and most importantly, lockdown to slow down the COVID 19 outbreak. The lockdown days differ by countries. Countries have set the days when the lockdown started and ended according to the COVID-19 effect on their public. Some countries have extended the lockdown by many days due to COVID-19 continues its influence intensely on the public. Chakraborty and Maity have emphasized that the lockdown has both environmental and economic impact on countries. The lockdown has created the ground for renewal of the environment, especially with the closure of factories and the reduction of both private and public transportation vehicles used. COVID-19 increased the air quality in many parts of the world with the lockdown imposed during the pandemic process [9]. Due to the lockdown, economic activities have stopped reducing carbon emissions [11].

To prevent this pandemic, governments have started to apply bans under many social restrictions. Lockdown is at the forefront of these restrictions. The aim of this study is to analyze statistically that the lockdown plays an important role in preventing COVID-19 and to show its psychological effect on people. This study used COVID-19 data from 49 countries to analyze the impact of the lockdown to slow down the COVID-19 outbreak. Countries that do not constantly enforce the lockdown are not included in this study. The correlation tests were used for data analysis based on unconstrained (normal) and constrained (Tukey-lambda).

This study includes five sections. The first section deals with the literature review of studies related to COVID-19 pandemic. The second part gives detailed information about the methodology of the study. The results obtained from the method mentioned in the methodology section are discussed in the third section. An overview of the psychological, environmental, and economic impacts of the lockdown imposed in countries due to COVID-19 is discussed in the fourth section. In the last section, conclusion about the study has been provided.

## 2 Methodology

COVID-19 case data of the countries considered were collected from www.worldometer.com [6]. A total number of 3726797 million confirmed active COVID-19 cases have been documented worldwide as of May 5, 2020. The number of approved active COVID-19 cases in countries considered for this study was recorded as 1440776 as of May 5, 2020. COVID-19 cases and lockdown days data were collected for 49 countries that implemented the lockdown between certain dates (without interruption). The lockdown days of the countries were obtained from the websites of the official institutions of each country.

The correlation test was used to analyze the associations between lockdown days factor and total cases of COVID-19 by countries. The correlation of the lockdown on the number of COVID-19 cases was calculated as unconstrained (normal) and unconstrained (Tukey-lambda distribution) in two ways. The distribution of Tukey-Lambda has the shape parameter λ. The Tukey-Lambda distribution is created with a position parameter, μ and a scale parameter, σ. This is because the general form of probability functions is expressed in terms of standard normal distribution. Values less than this mean (0.14) a heavy-tailed distribution (−1 is close to a Approx. Cauchy). That is, as the optimal value of λ increases from 0.14 to −1, progressively heavy tails are implied. Similarly, as the optimal value of λ becomes greater than 0.14, shorter tails are implied. The Tukey-lambda distribution is expressed mathematically in Eq. (1).

(1) $$G\left(p;\lambda \right)=\frac{p^{\lambda }-{\left(1-p\right)}^{\lambda }}{\lambda }$$

**Fig. 1:**
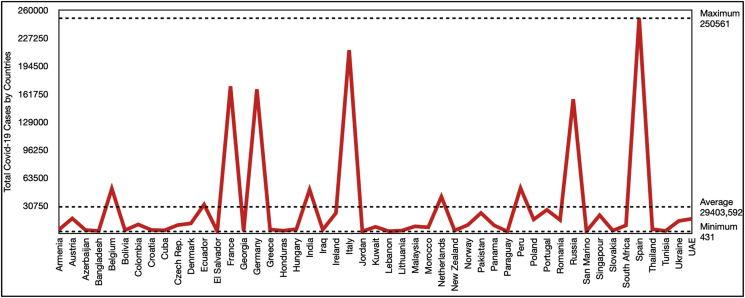
Cumulative confirmed COVID-19 cases by countries.

Fig. 1 shows the total number of COVID-19 cases by 49 countries. Most of the countries considered are located in the European region including Austria, Belgium, Denmark, France, Germany, Italy, Netherlands, Norway, Spain. Although COVID appeared in 19 China, the European region has become the epicenter of the virus, and more cases have emerged in Europe than in China. The highest case of COVID-19 from selected countries occurred in Spain, 250561 COVID-19 cases on May 5, 2020. Italy announced its first approved COVID-19 case on January 31, 2020.The country with the lowest COVID-19 case is Paraguay, 461 COVID-19 cases on May 5, 2020.

**Fig. 2:**
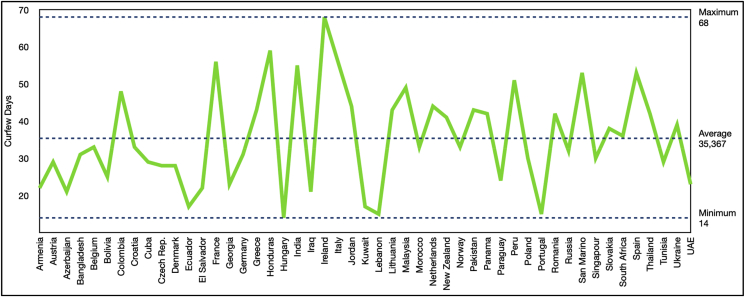
COVID-19 pandemic lockdown days by countries.

**Fig. 3:**
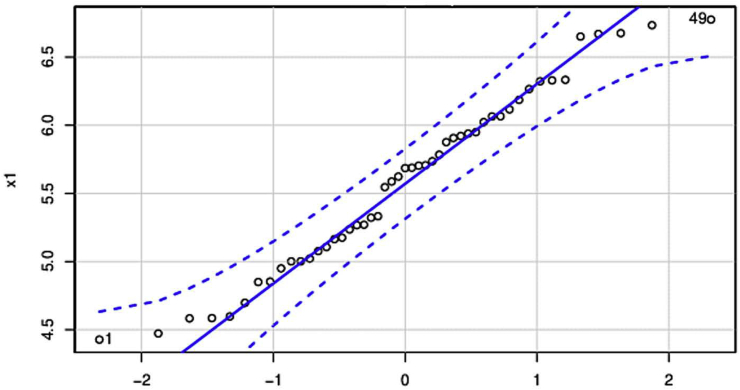
Transformed COVID-19 data.

Fig. 2 shows the days of lockdown imposed by 49 countries. Some of these countries continue the lockdown. However, the last day of lockdown in these countries was accepted as 5 May 2020 for this study. The Ireland, which has been curfewed for 68 days, has the longest lockdown period. A total of 21983 COVID-19 cases were approved as of May 5, 2020 in Ireland. Spain, the country with the highest number of cases, has been imposed lockdown for 53 days (see Fig. 3).

Although China became the center in the first days of the epidemic, Italy passed China with the emerging cases. Even though Italy suffered a severe injury in this pandemic, Italy have managed to control the number of COVID-19 cases with the lockdown for a long time. On the other hand, although there is a downward trend in new cases confirmed in France and Spain, the number of cases confirmed in Spain has exceeded the number of cases confirmed in Italy.

Descriptive analyses were implemented for all the data. The statistical test was two-sided, and a value p < 0.05 was measured for model and parameter statistically significant based on the fit regression model. The data used for the study were analyzed using JMP Pro software (version 15.0), Numbers and Minitab 18.0 statistical computer program.

## 3 Results and discussions

Descriptive analyses were presented for all the data used in this study in Table 1. The results of the descriptive analyses were prompted as 95% confidence intervals for upper and lower mean in lockdown days and total cases of COVID-19. The statistical test was two-sided, and a value *p* < 0.05 was measured for model and parameter statistically significant.

**Table 1:** Descriptive statistics data on Lockdown days and COVID-29 cases.

	Lockdown Days	Cases
Mean	35.367347	29403.592
Std Dev	13.080988	57887.549
Std Err Mean	1.8687126	8269.6499
Upper 95% Mean	39.124645	46030.837
Lower 95% Mean	31.610049	12776.346
N	49.000000	49.000000
Variance	171.11224	000003.35
Skewness	0.3677305	2.6740095
Kurtosis	−0.523178	6.3728059
Minimum	14.000000	431.00000
Maximum	68.000000	250561.00

The data set used is not suitable for normal distribution according to Anderson-Darling (the value of AD was 9.376 and p-value of Anderson-Darling test was 0.0003) and Shapiro-Wilk (the value of W was 0.728 and p-value of Shapiro-Wilk test was 0.010) normality tests. Statistical processes were performed by transforming COVID-19 data. The transformed COVID-19 data using full Box-Cox transformation method ($\text{for all}\;\lambda :\;\text{T}\left(\text{y}\right)\;=\;\left({\text{y}}^{\lambda }\;-\;1\right)\;/\;\lambda ,$ where the value of T(y) is the transformation of the observation data y; the value of λ shows the power to which all observation data to be increased) is limited between 4.4263 and 6.7749 to adapt to the normal distribution (see Fig. 1.).

**Fig. 4:**
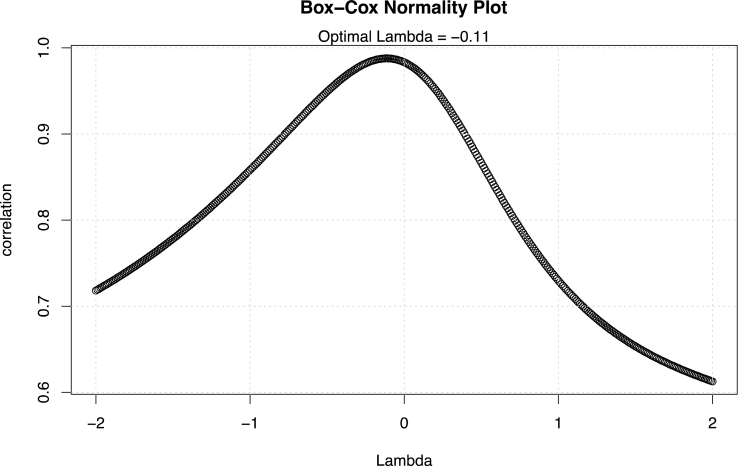
Tukey-lambda normality plot.

A correlation analysis was made between the spread of the COVID-19 pandemic and lockdown. The correlation value varies between −1 and +1. The correlation value of a factor indicates that it has a negative relationship as it approaches −1, and a positive relationship as it approaches +1. The lockdown has been found to have a very strong correlation on approved COVID-19 cases. The unconstrained correlation value was calculated as −0.9126. Fig. 4 shows the Tukey-Lambda correlation curve for normality test.

The Tukey-Lambda distribution forms a distribution family that can approach the normal distribution. The maximum correlation of lockdown and COVID 19 case numbers occurred for the λ value of 0.14 (r = 0.7402), and COVID-19 data was modeled according to a normal distribution (see Table 2).

**Table 2:** Tukey lambda-correlation.

Distribution	Lambda (λ)	Correlation (r)
Approx. Cauchy	−1.00	0.6557
Exact Logistic	0.00	0.7333
Approx. Normal	0.14	0.7402
U-Shaped	0.50	0.6968
Exactly Uniform	1.00	0.6697

The period of lockdown applied by 49 countries on average have taken 35.38 days. A lockdown was imposed for a minimum of 3 days while a lockdown was imposed for a maximum of 68 days by countries. During this period, an average of 29403 people in these countries were actively infected with COVID-19 virus. The number of confirmed COVID-19 cases was recorded as a minimum of 431 and a maximum of 250561. Predictive statistics of COVID-19 data and lockdown of the mentioned countries are given in Table 3. A minimum of 30 observations are required to create an effective statistical analysis. In this study, 49 data were used to calculate the lockdown in the aforementioned countries in the spread of COVID-19 pandemic with 95% relative confidence intervals (t-ratio = −0.83; F ratio = 5.7639; prob = 0.0413; adjusted R^2^ = 0.7212). It has been observed that the developed model was found important according to the statistical analyses. The lockdown parameter is significant at *p* < .05, so the data is very close to zero at 95.0% confidence level (t-ratio = 2.40; F ratio = 6.1614; prob = 0.0203).

**Table 3:** Validation of statistical analysis.

Source	Std Error	Sum of Squares	t-Ratio	F-Ratio	Prob > F Prob > |t|
Model	15944.03	19001496	−0.83	5.7639	0.0413
Lockdown Days	452.1786	6933250	2.40	6.1614	0.0203

The healthcare system capacities of countries have serious concerns about meeting the needs of infected COVID-19 patients. Therefore, countries have to take the strictest measures necessary to slow down or even stop this pandemic. Otherwise, this situation triggers the intensive care units to be at their maximum level in these countries. Although the number of infected patients is very high in Spain and Italy, the number of cases decreased significantly in recent days. This situation is also found in other countries. As a result of the strict measures taken, governments plan to return normal life gradually in the countries mentioned. As a result, an absolute decrease in the number of cases will occur if there is no possibility of virus mutation.

## 4 Effects of lockdown

### 4.1 Psychological effects

It is observed that there is a confusion with the rapid spread of the COVID-19 outbreak in the world and the emergence of serious consequences. For this reason, it is certain that the new data for COVID-19 mental health effects will be obtained more clearly with the big data to be obtained. According to the first findings obtained in the studies, Lockdown has been shown to be related to human psychology. It was determined that stress (8.0%) and depression (16.0–28.0%) were psychological reactions during the COVID-19 pandemic. These findings have some limitations. These psychological symptoms emerged from only a few of the affected countries and may not reflect the experiences of people living in other parts of the world. As a result, it is clear that having confirmed cases and mortality rates due to the COVID-19 pandemic has an impact on mental health problems.

### 4.2 Environmental effects

The effect of the lockdown on the environment due to Covid-19 has been addressed in many studies. It is observed that the environment has started to renew itself due to all kinds of industry, vehicle movement and social activities of people continue at a low level for a long time. In particular, a positive effect of lockdown restrictions on air and water quality has been observed. Yunus et al. have quantitatively determined that the quality of the water of Venbanad Lake has increased approximately 15.6% in India with the remote sensing imaging method [12]. Kerimray et al. have analyzed the effect of the 27-day lockdown in the city of Almaty, Kazakhstan on the concentrations of air pollutants, and emphasized the increase of air quality in Almaty [13]. Another study has showed that the quality of air due to the lockdown in Delhi has a positive effect [14]. Dantas et al. have calculated the CO emission level as approximately 30.3–48.5% due to the lockdown in Rio de Janeiro, Brazil [15]. For this study, we emphasized that the effect of lockdown on covid-19 was statistically significant. Examples of the environmental impacts of the indirect lockdown due to Covid-19 were provided.

### 4.3 Economic effects

The COVID-19 outbreak, which is now turning into a pandemic, is a global health crisis. However, the measures taken by countries against this epidemic bring along an unprecedented economic disaster [16]. The global pandemic, namely COVID-19, has been dealt with in many studies on the socio-economic effects of the world economy [17]. In almost 90% of the world, social isolation is applied in some way, people do not go out on the streets, workplaces are closed, flights are banned, people are dismissed. In terms of the extent of destruction in the economy during the pandemic and the speed of the expected recovery after the pandemic; at what level and when the outbreak will be brought under control, how long the current social distance/isolation-oriented measures will be loosened and when it will begin to normalize in the expansionary economic measures already taken.

## 5 The limitations of the study

There are some limitations of this study to measure the effect of Lockdown on COVID-19 cases. The COVID-19 pandemic is still ongoing so statistical analysis should continue. There are conflicting statements regarding lockdown by countries on COVID-19. In countries where the COVID-19 case is intensely occurring, either no lockdown is imposed or is applied intermittently. In addition, it is claimed that, besides the positive aspects of the lockdown, people who comply with this restriction cause a weakened immune system. The main reason for this is that there is too much food consumption and limited mobility. The effect of the lockdown caused by the COVID-19 pandemic on human health may be the subject of future work.

## 6 Conclusion

COVID-19's daily increasing cases and deaths have led to worldwide lockdown, quarantine and some restrictions. This study aims to analyze the effect of lockdown days on the spread of coronavirus in 49 countries. This study offers initial evidence that the COVID-19 pandemic can be suppressed by a lockdown. In addition, other parameters such as demographic of population, density of populations, the parameters of weather, economy, infrastructure of healthcare systems may be considered in the studies considering that it may be effective on COVID-19 pandemic. As a result, the application of lockdown by governments is also thought to be effective on psychology, environment and economy with it being effective on COVID-19.

## Ethical approval

Not applicable.

## Sources of funding

None.

## Author contribution

**Abdulkadir Atalan**: study design, data collections, data analysis, software, writing- original draft, writing-review & editing.

## Trial registry number

1.Name of the registry:

2.Unique Identifying number or registration ID:

3.Hyperlink to your specific registration (must be publicly accessible and will be checked):

## Guarantor

Dr. Abdulkadir Atalan.

## Provenance and peer review

Not commissioned, externally peer-reviewed.

## CRediT authorship contribution statement

**Abdulkadir Atalan:** Data curation, Formal analysis, Software, Writing - original draft, Writing - review & editing.

## Declaration of competing interest

None.
